# Effect of precursor amino acids for carnosine synthesis on breast fiber microstructures and myofiber differentiation-related gene expression in slow-growing chicken

**DOI:** 10.5713/ab.24.0012

**Published:** 2024-08-16

**Authors:** Cindy Riou, Panpradub Sinpru, Chanadda Suwanvichanee, Boonyarit Kamkrathok, Chalermluck Phoovasawat, Catleya Rojviriya, Wittawat Molee, Amonrat Molee

**Affiliations:** 1 School of Animal Technology and Innovation, Institute of Agricultural Technology, Suranaree University of Technology, Nakhon Ratchasima 30000, Thailand; 2 Synchrotron Light Research Institute (Public Organization), Nakhon Ratchasima, 30000, Thailand

**Keywords:** β-Alanine, Carnosine Content, Dietary Supplementation, L-Histidine, Meat Characteristics, Muscle Fiber Characteristics

## Abstract

**Objective:**

The effects of carnosine synthesis on the structural and microstructural determinants of meat quality have not been studied to date. Therefore, this study aimed to investigate the effect of supplementation with carnosine synthesis precursors on the characteristics and microstructure of breast muscle fibers in slow-growing Korat chickens (KR).

**Methods:**

Slow-growing KR were fed a non-supplemented commercial diet (control group) or a commercial diet supplemented with 1.0% β-alanine, 0.5% L-histidine, or a combination of both 1.0% β-alanine and 0.5% L-histidine. At 10 weeks, KR were slaughtered, and the breast muscle was collected. Samples were fixed and extracted to study the microstructure, fat level, and porosity of the meat using X-ray and scanning electron microscopy, and real-time polymerase chain reaction was performed to analyze the expression of genes related to myofiber differentiation.

**Results:**

L-histidine supplementation significantly altered myofiber diameter and muscle fiber density and compactness by regulating muscle fiber-type differentiation via carnosine synthase (*CARNS1*) and myocyte enhancer factor 2C expression, as well as myogenic differentiation antigen and myogenic regulatory factor 5 expression. While excess L-histidine potentially stimulated *CARNS1* to modify muscle fiber arrangement and tenderness in breast meat, dietary β-alanine supplementation alone or in combination with L-histidine supplementation induced a relatively less remarkable but not significant (p<0.05) effect on the breast meat characteristics studied.

**Conclusion:**

Interestingly, the combination of β-alanine and L-histidine supplementation had no effect on meat microstructure, meat porosity, and fat content in comparison with the control group. Thus, this combination had the best selectivity for improving meat quality. However, further studies are required to clarify the effects of carnosine levels on meat processing.

## INTRODUCTION

In Thailand, the slow-growing Korat chicken (KR) line, a crossbreed of a male Thai indigenous chicken (Leung Hang Khao) with the female broiler (Suranaree University of Technology), has been established to support smallholder farmers, ensure food security in communities, and preserve indigenous chicken breeds. KR meat shows a firmer and chewier texture along with a unique taste, lower fat content, and higher collagen content, resulting in higher sale prices than broiler meat [1].

Functional poultry meat is currently gaining consumer demand. The muscle fiber microstructure of meat has been reported to influence several aspects of meat quality, including its water-holding capacity, tenderness, texture, color, juiciness, flavor, and protein digestibility [2]. Carnosine is an endogenous dipeptide that is mostly found in the brain and skeletal muscles of vertebrates [3,4]. It is synthesized from β-alanine and L-histidine by carnosine synthase. Increased carnosine levels in chicken meat through genetic improvements can increase the value of chicken meat. Carnosine content in the breast meat of female native chickens has been reported to show low variation between lines [5]. Therefore, precursors of carnosine synthesis have been used to study the effects of increasing carnosine levels in chicken meat [6]. In previous experiments performed with the slow-growing KR line, the precursors of carnosine synthesis, β-alanine or L-histidine, were supplemented alone or in combination to the diet to stimulate the carnosine synthesis molecular pathway and investigate the pathways and effects of carnosine synthesis. In these experiments, supplementation of a combination of β-alanine and L-histidine was found to yield a higher carnosine meat content. Moreover, the highest carnosine levels did not affect meat quality by improving meat texture or changing secondary protein structures [6]. However, no previous reports have evaluated the levels of structural and microstructural changes in the muscle and the expression of genes related to carnosine function.

X-rays, which are a form of high-intensity electromagnetic radiation, have excellent investigative value and penetrating ability, and are unconstrained by the morphological convolution of certain materials. X-ray tomography is steadily becoming an important tool for studying the microstructure of foods [7]. Synchrotron radiation X-ray tomographic microscopy (SRXTM) is a nondestructive technique for visualization and analysis of the internal features of solid opaque objects. It can enable accurate three-dimensional (3D) reconstruction of internal structures by recording the differences in the effects of the passage of energy waves that react with those structures. Synchrotron, a type of particle accelerator, is a powerful monochromatic X-ray source, and synchrotron-based radiography is a sensitive approach for producing tomographic data with exceptional clarity and resolution [8]. Therefore, the SRXTM technique is expected to produce significant results in the microstructural analysis of KR meat.

Myofiber properties play a crucial role in influencing meat tenderness [9]. Type I and II myofibers, called slow- and fast-twitch fibers, respectively, differ in their contractile or endurance capacities, metabolic capabilities, and ultrastructural morphology [10]. Myofiber differentiation is regulated by a signaling pathway involving calcineurin [10] and calcium- and calmodulin-dependent serine/threonine protein phosphatases [11]. Calcineurin activation can upregulate slow-fiber-specific promoters and induce fast-to-slow fiber transformations [10]. However, reports describing the effects of carnosine synthesis on the structural and microstructural determinants of meat tenderness and the expression of genes related to the calcineurin pathway, such as calcineurin (*CaN*), calmodulin (*CaM*), nuclear factor of activated T-cells (*NFATc1*), peroxisome proliferator-activated receptor-γ coactivator-1-α (*PCG1-α*), myocyte enhancer factor 2C (*MEF2C*), myogenic differentiation antigen (*MyoD*), and myogenic regulatory factor 5 (*Myf5*), in slow-growing chicken, particularly when the body of chicken is stimulated to synthesize carnosine, are limited.

Therefore, we aimed to investigate the effect of supplementation of the carnosine synthesis precursors, 1% β-alanine and 0.5% L-histidine, which have been shown to significantly increase the carnosine content in chicken breast meat without affecting lipid oxidation, on the characteristics and microstructure of muscle fibers in slow-growing chickens. We expect that the insights gained from this study will provide a precise direction for breeding programs to improve the genetics of chicken as a source of carnosine.

## MATERIALS AND METHODS

### Ethics approval

The experiments were performed in accordance with the Animal Care and Use Committee Guidelines of the Suranaree University of Technology, Nakhon Ratchasima, Thailand; document ID: 18/2560.

### Experimental animals

A total of 1,900 one-day-old female KR chicks from the Suranaree University of Technology farm were used in this study. They were reared at the Poultry Research Unit of the University Farm, Suranaree University of Technology and fed a commercial diet (CPF Co., Ltd., Nakhon Ratchasima, Thailand) and water ad libitum. At three weeks of age, 400 female KR chickens were randomly divided into four experimental groups. Each experimental group consisted of five replicates (20 birds per replicate). The chickens were fed a commercial diet that was not supplemented (control group, n = 100) or supplemented with 1.0% β-alanine (n = 100), 0.5% L-histidine (n = 100), or a combination of 1.0% β-alanine and 0.5% L-histidine (n = 100). The animals had free access to food and water during the treatment period. The amino acid contents of the diets are presented in Supplementary Table S1.

### Sample collection

At 10 weeks of age, 20 birds (one chicken per replicate from each group) were randomly slaughtered using chloroform stunning for breast muscle collection. Data on growth performance and muscle carnosine and anserine content are shown in Supplementary Tables S2 and S3, respectively. Samples were excised in cross-sections (1×1×3 mm) from the central area of the pectoralis major muscle using a scalpel. One portion of each collected sample was fixed in 10% formalin to study the microstructure and porosity of the meat. The other part was immediately frozen and stored at –80°C to analyze the fat content and the expression of genes related to myofiber differentiation.

### Sample preparation for microstructural and porosity analysis

The samples were rinsed with water to remove formalin and dehydrated in a series of 50%, 70%, 90%, and 100% ethanol solutions at 30-min intervals. For microstructural analysis, samples were placed in a Leica EM CPD300 critical point dryer (Leica Microsystems, Wetzlar, Germany) for 1 h to replace ethanol with CO_2_, and then placed overnight in a C-3W Dry Keeper auto-desiccator (Sanplatec Corp., Osaka, Japan) to maintain CO_2_ within the tissue. For porosity analysis, the samples were air-dried overnight.

### Microstructural analysis

Sample fragments (three samples per group) were mounted on aluminum stubs, coated with gold (3 min), and observed using a JSM-6010LV scanning electron microscope (SEM; JEOL Ltd., Tokyo, Japan). Micrographs of the meat cross-sections were taken at a magnification of 1,000×, and three views were captured for each section. The number of myofibers per section, myofiber diameter, distance between myofibers, percentage of myofiber area, and spacing were measured using ImageJ software v.1.8.0 (National Institutes of Health, Bethesda, MD, USA).

### Porosity and fat content analysis

SRXTM was used to analyze the porosity and fat content of chicken meat. Meat samples obtained from each chicken (three samples per group) were cut into cubes (3×3×3 mm). For the porosity analysis, meat samples were air-dried overnight prior to the SRXTM experiment. For fat analysis, the samples were thawed at 4°C overnight. All SRXTM experiments were performed at a Beamline 1.2W: X-ray tomographic microscopy, Siam Photon Source (SPS) facility, Synchrotron Light Research Institute (Public Organization), Nakhon Ratchasima, Thailand. Synchrotron radiation was generated using a 2.2-Tesla multipole wiggler at the SPS.

For X-ray tomography data collection, the sample was stabilized in a polyimide tube sample holder mounted on a rotary stage 32 m from the radiation source. A total of 1,800 X-ray projections were obtained for rotations of 0° to 180° with an angular increment of 0.1°. X-ray imaging was performed using a filtered polychromatic X-ray beam (mean energy, 10 keV). All X-ray projections were acquired at a pixel size of 1.44 μm in a 16-bit dynamic range of greyscale, obtained using a detection system equipped with a 200-μm-thick YAG:Ce scintillator, a 5× objective lens-coupled microscope (Optique Peter, Lentilly, France), and a PCO.edge 5.5 sCMOS camera (PCO AG, Kelheim, Germany). After data collection, the X-ray projections were normalized using flat-field correction with open-beam and dark images. Tomographic reconstruction was performed using the filtered back-projection algorithm with Octopus Reconstruction software [12].

The porosity of air-dried samples and the fat content of fresh samples were analyzed from the reconstructed images using Octopus Analysis software [12]. The density variation of air, fat, and meat contributed to the differential X-ray absorption, which allowed for segmentation analysis of the voids. The voids and fat were segmented by binary thresholding of the grayscale degree. Porosity and fat content were calculated for an analyzed volume of 0.432 mm×0.288 mm ×0.145 mm. Volume presentation of the porosity and fat content distributed in the analyzed volumes was made using Octopus Visualization software [12].

### Real-time polymerase chain reaction analysis

Total RNA was extracted from five chickens per group using TRIzol reagent (Thermo Fisher Scientific, Carlsbad, CA, USA). The breast tissue from each chicken was lysed and homogenized using TRIzol reagent. After centrifugation using Universal 320 R (Thermo Fisher Scientific, Langenselbold, Germany) at 12,000×*g* and 4°C for 10 min, the supernatants were transferred to new tubes and incubated with chloroform for 5 min. Samples were then centrifuged for 10 min at 12,000×*g*. Pellets were precipitated using isopropanol, washed with 75% ethanol, and dried at 25°C for 5 min. RNA pellets were resuspended in 20 μL of nuclease-free water. A NanoDrop 2000 (Thermo Fisher Scientific, Waltham, MA, USA) instrument and 1% agarose gel were used to analyze the concentration and quality of the extracted RNA. The expression levels of carnosine synthase (*CARNS1*), *CaN*, and other genes related to the calcineurin pathway (*CaM*, *NFATc1*, *PCG1-α*, *MEF2C*, *MyoD*, and *Myf5*) were measured by quantitative-reverse transcription polymerase chain reaction (qRT-PCR) using previously published primers [9]. Total RNA was reverse-transcribed into first-strand cDNA using a High-Capacity cDNA Reverse Transcription Kit (Thermo Fisher Scientific, San Mateo, CA, USA). The amplification efficiency of each primer pair was calculated before qRT-PCR analysis. qRT-PCR was performed in triplicate on a LightCycler 480 Real-Time PCR System (Roche Diagnostics GmbH, Mannheim, Germany) using PowerUp SYBR Green Master Mix (Thermo Fisher Scientific, Vilnius, Lithuania). The thermocycling program consisted of an initial denaturation step at 95°C for 5 min, followed by 40 cycles of 95°C for 10 s, 60°C for 30 s, and 72°C for 30 s, with a final extension at 72°C for 5 min. Relative gene expression was calculated using the 2^–ΔΔCt^ method, using the housekeeping gene 18s as an internal control.

### Principal component analysis

The differences and similarities among the four experimental groups were evaluated via principal component analysis (PCA) of all studied variables (microstructural parameters, meat porosity, fat content, and carnosine- and calcineurin-related gene expression) using the ropls R package [13].

### Statistical analysis

All data were subjected to one-way analysis of variance (ANOVA) using GraphPad Prism v.9.2.0 statistical software (GraphPad Software Inc., San Diego, CA, USA), and Tukey’s test was used to perform pairwise comparisons of the means. Pearson’s correlation coefficient values were calculated to examine the relationships among myofiber bundle characteristics. Statistical significance was set at p<0.05.

## RESULTS

### Growth performance traits

Growth performance data for the KR line have been previously reported by Suwanvichanee et al [6]. The treatment groups showed no significant differences in growth performance.

### Meat microstructure

**Figure 1:**
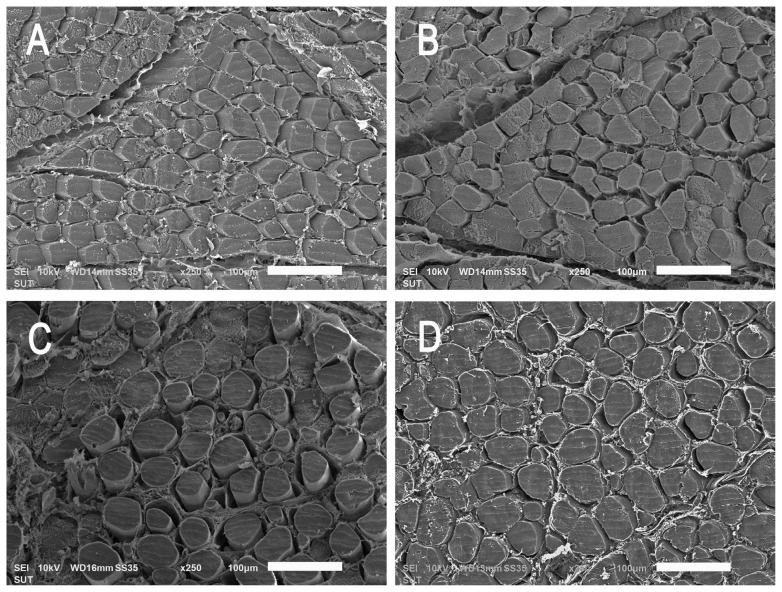
Scanning electron micrographs of meat cross-sections from Korat chickens (KR) (A) that did not receive supplementation (control), or were (B) supplemented with 1.0% β-alanine and 0.5% L-histidine, (C) 1.0% β-alanine alone, or (D) 0.5% L-histidine alone. Scale bar indicates 100 μm, magnification = 250×.

**Figure 2:**
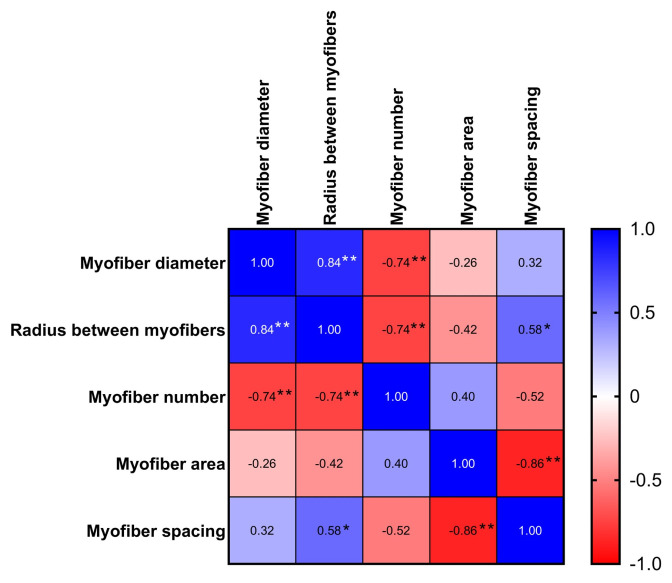
Correlation matrix of myofiber bundle characteristics. Blue and red boxes indicate positive and negative correlations, respectively. Deeper shades of blue or red represent relatively higher correlations. Pearson correlation analyses were performed between all variables (** p<0.01, * p<0.05).

The effects of dietary supplementation of the carnosine synthesis precursors β-alanine and/or L-histidine on breast meat microstructure are shown in Figure 1 and Table 1. The myofiber diameter of the group that received L-histidine alone was higher than those of the β-alanine + L-histidine, β-alanine alone, and control groups. The differences in the cross-sections of the pectoralis major muscles revealed that the myofibers in the control group were closely packed (Figure 1A), indicating a compact arrangement, whereas the myofibers in birds supplemented with β-alanine and/or L-histidine showed a few gaps (Figure 1B). In birds supplemented with β-alanine or L-histidine alone, the myofiber arrangement was scattered and numerous gaps were observed (Figure 1C and 1D). The correlations among myofiber bundle characteristics are reported in Figure 2. An increase in myofiber diameter was negatively correlated with the number of myofibers per cross-section (p<0.01) and positively correlated with the distance between myofibers (p< 0.01). An increase in the distance between myofibers was positively correlated with myofiber spacing (p<0.05) and negatively correlated with myofiber number (p<0.01). Thus, L-histidine supplementation alone caused an increase in breast diameter, negatively affecting muscle fiber density and compactness. However, supplementation with β-alanine alone or in combination with L-histidine had no effect on myofiber diameter.

### Meat porosity and fat content

**Figure 3:**
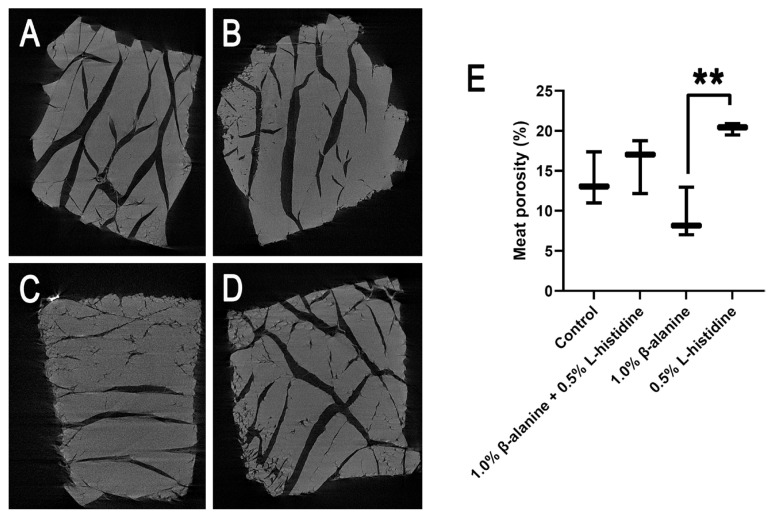
Effect of supplementation with β-alanine and/or L-histidine on Korat chicken (KR) meat porosity. Reconstructed two-dimensional cross-sections of KR breast muscle from (A) control birds and (B) birds supplemented with 1.0% β-alanine and 0.5% L-histidine, (C) 1.0% β-alanine alone, or (D) 0.5% L-histidine alone. (E) Boxplot of the meat porosity percentage per treatment. The experiment was performed with three biological replicates per group, and the p-value was assessed via one-way analysis of variance (** p<0.01).

**Figure 4:**
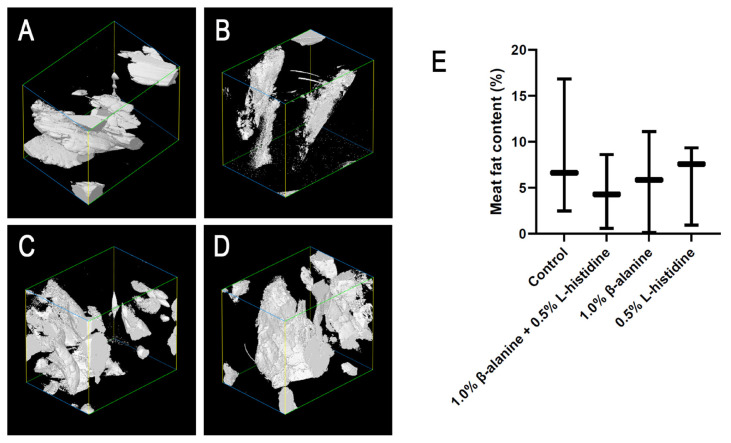
Meat fat content in Korat chicken (KR) supplemented with 1.0% β-alanine and/or 0.5% L-histidine. Three-dimensional reconstruction of the fat content in breast muscle samples from (A) control birds or (B) birds supplemented with 1.0% β-alanine and 0.5% L-histidine, (C) 1.0% β-alanine alone, and (D) 0.5% L-histidine alone. (E) Boxplot of meat fat content percentage per treatment. The experiment was performed in triplicate per group, and evaluated via one-way analysis of variance (p<0.05).

The reconstructed cross-sectional images obtained by X-ray tomographic microscopy revealed different meat porosities among the four groups (Figure 3). The pores distributed in the meat were identified and segmented on the basis of the contrast in density between air and meat. Large irregular black voids corresponding to cracks and fractures were observed in all groups, particularly in the L-histidine-supplemented group (Figure 3D). The void area in the group that received β-alanine supplementation alone was smaller than those in the control group and the groups that received L-histidine alone and both β-alanine and L-histidine (Figure 3A–3D). Moreover, the meat porosity in the group that received supplementation with L-histidine alone was significantly (p<0.05) higher than that in the groups supplemented with β-alanine (Figure 3E). The fat content in breast meat was low in the group that received both β-alanine and L-histidine supplementation and higher in the control group and the groups that received β-alanine or L-histidine supplementation alone (Figure 4A–4D). However, no significant differences were observed among the groups (Figure 4E). These results showed that supplementation with β-alanine and/or L-histidine had no effect on meat porosity and fat content.

### Carnosine synthase expression

**Figure 5:**
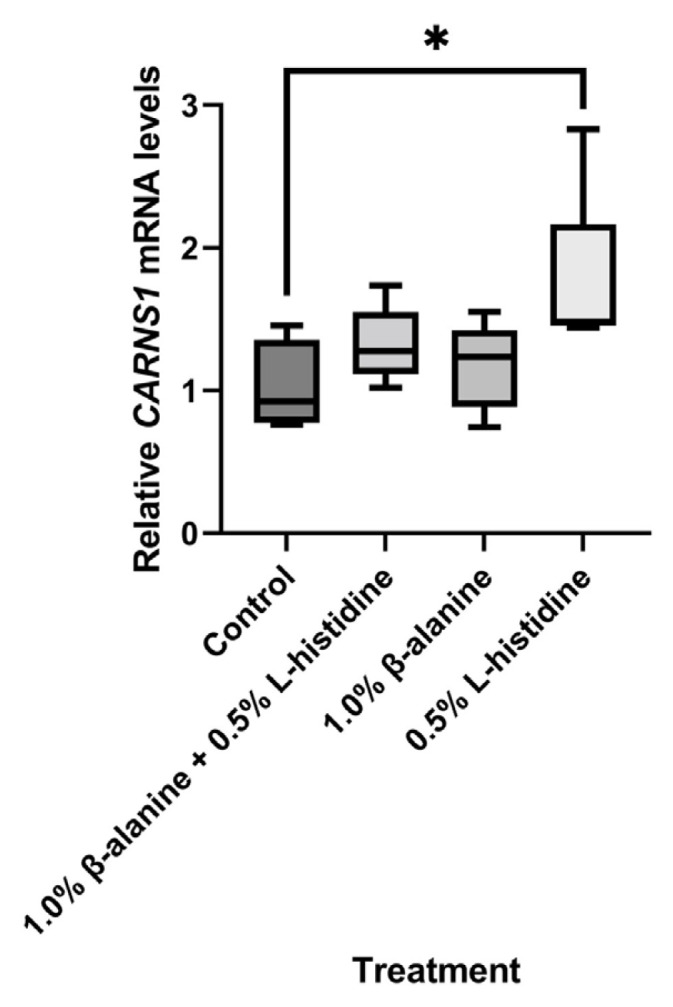
Effect of supplementation with β-alanine and/or L-histidine on carnosine synthase expression in breast muscle in Korat chicken. The experiment was performed using five biological replicates per group and three technical replicates per individual, and the p-values were assessed by one-way analysis of variance (* p<0.05). CARNS1, carnosine synthase 1.

The effect of dietary β-alanine and/or L-histidine supplementation on carnosine synthase expression in the breast revealed that the *CARNS1* mRNA level in the L-histidine supplementation group was significantly greater (p<0.05) than that in the control group (Figure 5). However, supplementation with β-alanine alone or in combination with L-histidine supplementation had no significant effect on the *CARNS1* mRNA level. Thus, the increased *CARNS1* expression in the group supplemented with L-histidine may have been affected by the functional control of the body after supplementation.

### Calcineurin pathway gene expression

**Figure 6:**
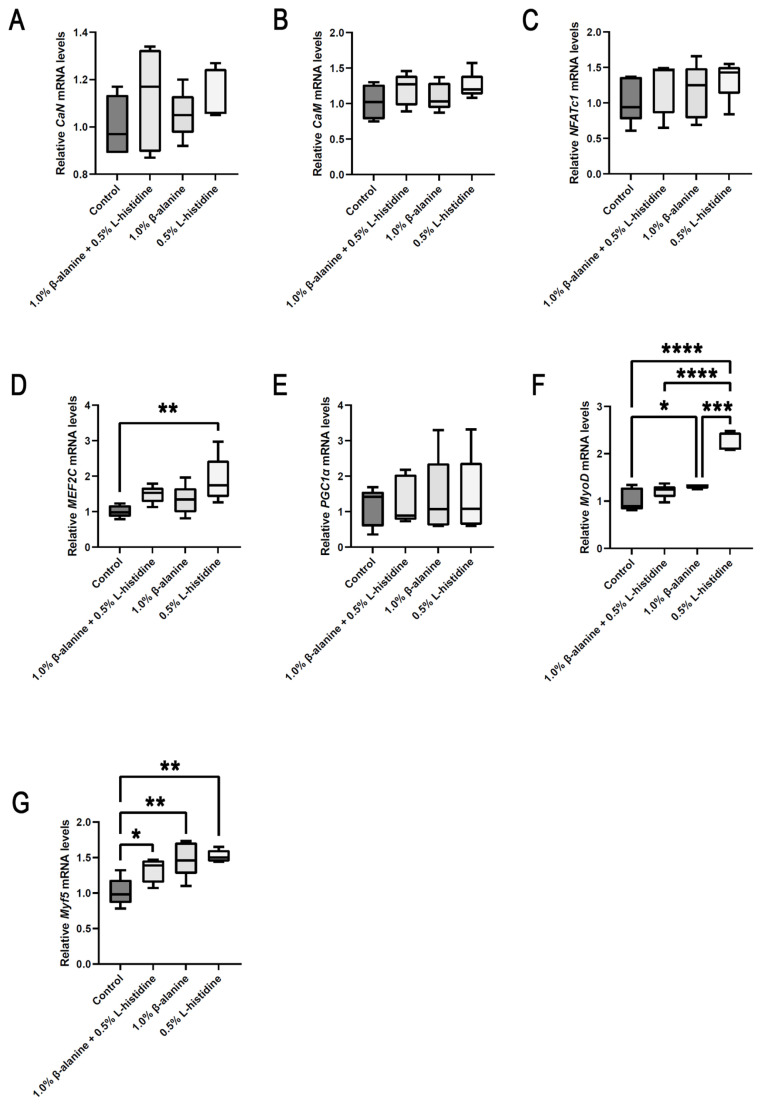
Effects of β-alanine and L-histidine supplementation on the expression of calcineurin-related genes in the breast muscle of Korat chicken. The experiment was performed in five biological replicates per group and three technical replicates per individual, and the p-values were assessed by one-way analysis of variance (* p<0.05, ** p<0.01, *** p<0.001, **** p<0.0001). *CaN*, calcineurin; *CaM*, calmodulin; *NFATc1*, nuclear factor of activated T-cells; *MEF2C*, myocyte enhancer factor 2C; PGC-1α, peroxisome proliferator-activated receptor-γ coactivator-1-α; *MyoD*, myogenic differentiation antigen; *Myf5*, myogenic regulatory factor 5.

Our analyses of the effects of β-alanine and/or L-histidine supplementation on calcineurin-related gene expression in breast muscle showed that the relative levels of *CaN*, *CaM*, *NFATc1*, and *PGC-1α* mRNA were not different among groups (Figure 6A–6C, 6E). In comparison with the control group, the group that received L-histidine supplementation showed significant differences in the expression of *MEF2C*, *MyoD*, and *Myf5* (Figure 6D, 6F, and 6G). Supplementation with β-alanine caused significant expression of *MyoD* and *Myf5* (Figure 6F and 6G). In addition, β-alanine and L-histidine supplementation caused a significant increase in *Myf5* expression levels (Figure 6G). These findings indicated that significant changes in gene expression were involved in the changes in the muscle myofibers of KR.

### Analysis of the relationship between *CARNS1* expression, calcineurin-related gene expression, and myofiber bundle characteristics

**Figure 7:**
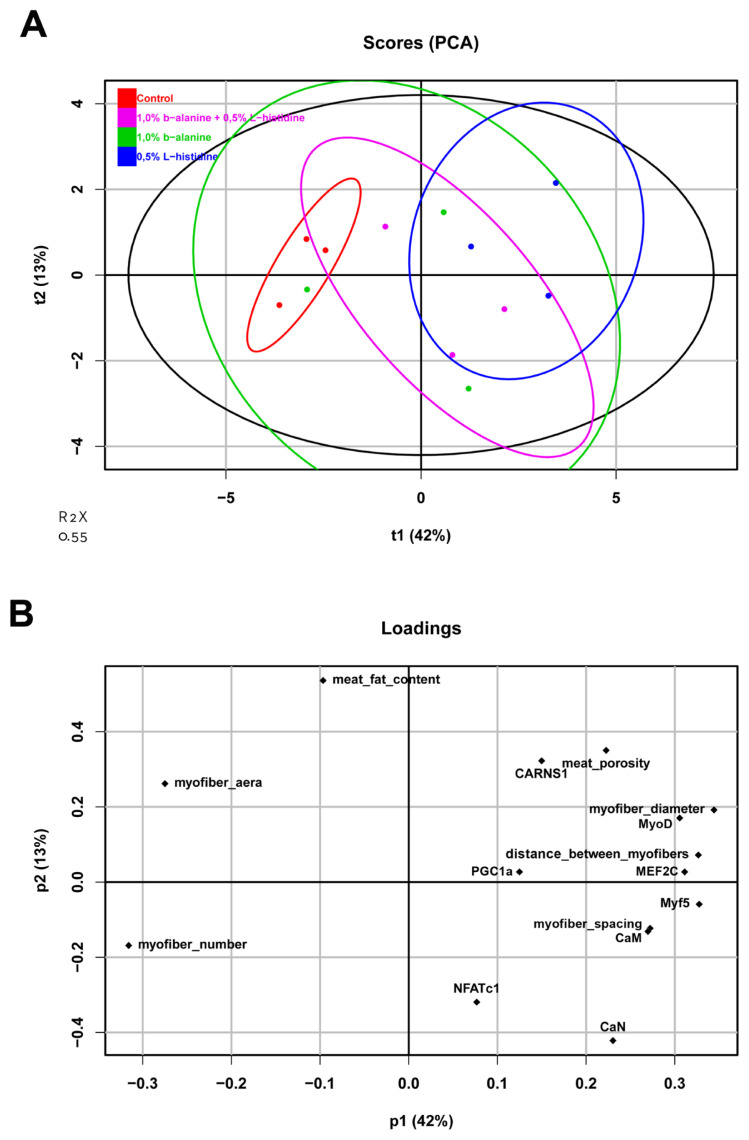
(A) Principal component analysis and (B) loading plots of all variables of Korat chicken breast after supplementation with 1.0% β-alanine and 0.5% L-histidine, 1.0% β-alanine alone, 0.5% L-histidine alone, or without supplementation (control). In each plot, the abscissa and ordinate each represent a principal component, and the percentage represents the amount of variation between samples. R2X represents the accumulated variance contribution rate. PCA, principal component analysis.

The PCA revealed a clear separation between the control and L-histidine-supplemented groups (Figure 7A). The PCA showed that 42% of the total variation was explained by the first principal component (p1) and 13% by the second principal component (p2).

The loading plot revealed that p1 was mainly defined by the myofiber number, myofiber diameter, distance between myofibers, and expression of *MyoD*, *MEF2C*, and *Myf5* (Figure 7B). The myofiber number variable was located opposite the variables for myofiber diameter, distance between myofibers, and *MEF2C* and *MyoD* expression, indicating a negative relationship among these variables. In contrast, p2 was defined by the meat fat content, meat porosity, and *CARNS1*, *NFATc1*, and *CaN* expression. The opposite location of the meat fat content and the expression of *NFATc1* and *CaN* indicated that the expression of these genes had a negative relationship with meat fat content. Based on their locations on the same side of the plot, *CARNS1* expression was considered to be positively associated with meat porosity, myofiber diameter, distance between myofibers, and *CaN*, *NFATc1*, *MEF2C*, *MyoD*, and *Myf5* expression. In contrast to the control group, the group supplemented with L-histidine had the highest positive p1 and p2 values, which showed the highest values for meat porosity, myofiber diameter, distance between myofibers, and *CARNS1*, *MEF2C*, *MyoD*, and *Myf5* expression. These results clearly show the differences and similarities between the experimental groups and the associations of all studied variables.

## DISCUSSION

This is the first study to reveal the effects of supplementation with carnosine synthesis precursors on muscle fiber microstructure and the expression of genes that regulate carnosine synthesis and myofiber differentiation in the breast muscle of slow-growing chickens.

KR meat has a distinct taste. Our previous study revealed that supplementation with β-alanine and/or L-histidine increased the carnosine levels of KR meat. However, meat quality was unaffected by high carnosine levels [6]. The results of this study demonstrate the effects of supplementation with carnosine synthesis precursors on breast meat microstructure. All groups supplemented with β-alanine and/or L-histidine showed greater breast myofiber diameters than the control group. The group that received L-histidine supplementation alone showed a significant difference in comparison with the control group. An increase in myofiber diameter was negatively correlated with muscle fiber density and compactness, indicating a minor modification of meat texture. Thus, L-histidine supplementation negatively affected KR meat quality. The results from a previous study revealed that dietary supplementation with 0.5% L-histidine combined with 1.0% β-alanine significantly increases carnosine levels, which are correlated with increasing pH_45 min_ and decreasing drip loss, cooking loss, shear force, and lipid oxidation, suggesting that carnosine has a positive effect on KR meat texture [6]. In the group supplemented with both β-alanine and L-histidine in the present study, the diameter did not differ from that in the control group, resulting in unchanged KR meat quality. However, Cong et al [9] reported that changes in muscle fiber characteristics coincide with positive improvements in meat quality. Dietary carnosine supplementation increases muscle fiber density and decreases muscle fiber diameter, shear force, and hardness (improves meat tenderness). A small diameter and high density of muscle fibers has been reported to explain enhanced tenderness in pig meat [14]. Thus, meat quality and microstructure are important factors that can be influenced through genetic improvement. Our previous study reported that supplementation with β-alanine and L-histidine resulted in the highest levels of carnosine in KR meat [6]. Taken together, these findings imply that combined β-alanine and L-histidine supplementation did not affect meat microstructure.

In this study, meat porosity increased with L-histidine supplementation and decreased in the combined β-alanine/L-histidine, control, and β-alanine groups. In addition, while the group receiving L-histidine supplementation alone showed a significantly higher difference in meat porosity than the groups receiving β-alanine, no difference was observed in the control group, indicating that meat texture does not change with supplementation of carnosine synthesis precursors. Notably, supplementation with L-histidine, not β-alanine, has been reported to improve the water-holding capacity of KR breast muscle because it induces a significant decrease in drip loss [6], demonstrating that high carnosine content improves the water-holding capacity of KR meat. Yang et al [15] reported that high carnosine content is associated with a high water-holding capacity in pigs. Porosity is a particularly important physical parameter for meat quality because it influences the moisture content and rehydration potential of meat [16]. Muscles constitute approximately 75% of the water content, which is held by capillary forces and surface tension [17]. Porosity and pore structure are important factors that affect water diffusion. In addition to showing the relatively higher expression of *CARNS1* in the L-histidine-supplemented group, our data implied that dietary L-histidine supplementation may stimulate muscle modification due to an increase in pore size, which may impact the water-holding properties of the meat. Further studies on pore structure can provide a better understanding of the relationship between porosity and water-holding capacity in slow-growing chicken breasts.

In terms of fat content, intramuscular fat (IMF) is a key indicator of meat quality and has a positive impact on meat appearance, tenderness, and flavor. In the present study, the IMF content varied among individuals and showed no significant difference in relation to β-alanine and/or L-histidine supplementation. These results are consistent with the findings of our previous studies. Synchrotron radiation-based Fourier transform infrared microspectroscopy revealed that the percentage of lipid integration did not differ significantly between the supplemented and control groups. L-Carnosine supplementation has been shown to not affect IMF content in broiler breast muscle [18]. Although IMF-related genes have been recently reported to be differentially expressed in KR breast muscle between the β-alanine- and L-histidine-supplemented groups (Acyl-CoA synthetase bubblegum family member-1), the effect of these genes on IMF content is still unclear [19]. Therefore, we conclude that supplementation with β-alanine and L-histidine, which are carnosine synthesis substrates, may not affect the IMF content and adipogenesis in KR breast muscle.

*CARNS1* encodes carnosine synthase 1, which catalyzes the endogenous synthesis of carnosine from β-alanine and L-histidine [15]. Fast-twitch type II muscle fibers have higher carnosine concentrations than slow-twitch type I fibers [20]. The high carnosine content in type II fibers is due to the fact that the muscles buffer protons in these fibers with the highest rate of carboxylate anion production [21]. However, excess histidine is associated with oxidative stability [22], which, in turn, affects cellular homeostasis [23]. Consistent with a previous study [24], we found that L-histidine supplementation increased *CARNS1* expression in KR breast muscle, indicating that L-histidine supplementation can stimulate carnosine production in KR breast. Therefore, *CARNS1* expression may increase to prevent the adverse effects of histidine via carnosine synthesis. In contrast to previous studies [25,26], we found that *CARNS1* mRNA level was not significantly increased by β-alanine supplementation alone or in combination with L-histidine when compared with non-supplementation. β-Alanine supplementation in older broiler chickens has been shown to have no effect on the carnosine content in muscle [27]. In addition, ATP-grasp domain-containing protein 1 expression in slow-growing chickens is downregulated with age, suggesting that the stimulation of *CARNS1* expression by increasing β-alanine levels depends on chicken age. In the present study, we observed a slight increase in *CARNS1* expression after supplementation with a combination of β-alanine and L-histidine. Suwanvichanee et al [6] reported that supplementation with both amino acids (β-alanine and L-histidine) increases the highest carnosine concentration by 52.8% in KR meat in comparison with the concentration in the group that did not receive supplementation. Qi et al [24] found that *CARNS1* expression did not increase significantly when carnosine content increased, suggesting that an increase in carnosine content may not be directly related to increased *CARNS1* expression. Moreover, carnosine production has been reported to depend on the combination of supplemented L-histidine and available β-alanine in the blood and muscle [28]. This may imply that increased *CARNS1* expression in animals is a part of mechanisms to control the L-histidine content balance in body.

In the current study, analyses of the effects of supplementation with carnosine synthesis precursors on calcineurin-related genes revealed that *CaN* and *CaM* expression were not altered by β-alanine and/or L-histidine supplementation in KR breast muscle. A previous study on chicken muscle showed that the expression of *CaN* and *CaM* is associated with adipogenesis [29]. CaN regulates adipocyte differentiation by inhibiting the expression of adipocyte differentiation-associated transcription factors [30]. In our study, consistent with the findings for *CaN* and *CaM* expression, no difference was observed in the fat content of breast muscle among the groups. In contrast, in broiler chicken thighs, which are mainly composed of slow-twitch type I fibers and IMF, dietary carnosine supplementation increased *CaN* and *CaM* expression [9]. *CaN* can selectively upregulate the promoter activity of slow-twitch fibers depending on the calcium concentration [10]. However, the IMF content in the thigh is one of the main factors leading to lipid oxidation [31]. Increased lipid oxidation causes changes in cell signaling pathways, affecting the release of calcium from the sarcoplasmic reticulum and resulting in damage to the contractile capacity of muscle cells [32]. Carnosine and histidine directly or indirectly regulate the activity of calcium ion channels in the skeletal muscle sarcoplasmic reticulum to maintain muscle contraction performance [33]. Consistent with the results for IMF, we suggest that CaN and CaM in the breast of KR, which have low IMF content, may be activated to decrease the influence of lipid oxidation in muscle cells via carnosine synthesis.

Among the known substrates of CaN in muscle, members of the nuclear factor of activated T-cells (NFAT) family and those of the myocyte enhancer-binding factor (MEF2) family of transcription factors are the most well-characterized [10]. Despite a slight increase in *NFATc1* expression in all supplementation groups, our results showed that *NFATc1* expression was not significantly regulated by supplementation with dietary β-alanine alone or in combination with L-histidine. However, *MEF2C* expression was significantly upregulated in the L-histidine-supplemented group. In contrast, in the thighs of broiler chickens supplemented with dietary carnosine, *NFATc1* expression is upregulated and *MEF2C* expression was not regulated [9]. NFATc1 has been reported to control the differentiation of skeletal muscle fiber types and is required for the fast-to-slow fiber-type transition via direct inhibition of MyoD [34]. A recent study showed that MEF2C is an essential regulator of the microtranscriptome during skeletal muscle differentiation [35]. MEF2C is an essential partner of myogenic factors such as MyoD in activating transcription and myogenesis [36]. Our data indicate that L-histidine supplementation alone, not β-alanine supplementation alone or combined L-histidine and β-alanine supplementation, affects the regulation of the breast muscle fiber-type transition via *MEF2C* expression.

In the present study, dietary supplementation with L-histidine and β-alanine significantly increased MyoD expression in comparison with that in the control group. Interestingly, *Myf5* expression in the KR breast muscle was upregulated in all supplementation groups. *MyoD* and *Myf5* are expressed in muscle stem cells, also known as satellite cells [37]. These genes are considered specific markers of satellite cells and are used to identify proliferating satellite cells that participate in muscle fiber regeneration [38]. A previous study on slow-growing chickens showed that the expression of *MyoD* and *Myf5* in muscle satellite cells is related to muscle growth rate [37]. In addition, our results showed that *MyoD* expression was correlated with *CARNS1* expression. Notably, MyoD accumulation has been shown to differ between muscle fiber types I and II [38]. The hypothesis is that the activation of MEF2C, MyoD, and CARNS1 in the KR breast muscle appears to be a protective compensatory response to maintain muscular performance from dietary supplementation with L-histidine and/or β-alanine.

Consistent with the effect of dietary supplementation on calcineurin expression, we found that dietary β-alanine and L-histidine supplementation had no effect on *PGC1-α* expression. PGC-1α has been reported to be a target of the calcineurin signaling pathway [39]. PGC-1α plays a key role in regulating the switching and determination of muscle fiber types, and it is preferentially expressed in muscles enriched in type I fibers [39]. The role of PGC-1α in the skeletal muscles of broiler chickens has been recently explored *in vitro*, and *PGC-1α* overexpression has been suggested to facilitate skeletal muscle type I fiber formation [40]. *PGC-1α* expression is correlated with the expression of *MyoD*, *MEF2C*, and other key genes related to skeletal myocyte differentiation [40]. However, our findings suggested that PGC-1α may not be considerably activated by β-alanine and L-histidine supplementation in the regulation of muscle fiber type in KR breast muscle.

The increased meat porosity and expression of *CARNS1*, *MEF2C*, *MyoD*, and *Myf5* in the dietary L-histidine supplementation group led to an increase in the diameter of myofibers and, consequently, modified muscle fiber arrangement to create greater spacing between myofibers, suggesting that transformation of muscle fiber types may improve the oxidative status and oxidative stability of muscle cells. Dietary β-alanine supplementation alone or in combination with L-histidine may have a less remarkable impact on meat texture modification because it is unaffected by overabundant histidine (combination with carnosine). Therefore, increasing carnosine synthesis may help maintain muscle contraction performance due to oxidative stability imbalance, leading to the modification of muscle fiber arrangement and a consequent improvement in meat tenderness. These changes in meat porosity reflected an important difference between the effects of L-histidine and/or β-alanine supplementation.

## CONCLUSION

The present study is the first to characterize the effects of β-alanine and L-histidine supplementation on KR chicken meat characteristics, meat microstructure, and the regulation of muscle fiber-type differentiation. We demonstrated that L-histidine supplementation increases myofiber diameter, muscle fiber density, and compactness and affects the regulation of muscle fiber-type differentiation via *CARNS1*, *MEF2C*, *MyoD*, and *Myf5* expression Thus, CARNS1 may be stimulated by excess L-histidine, modifying muscle fiber arrangement and tenderness in breast meat. In contrast, β-alanine supplementation had a relatively less remarkable effect on the characteristics of breast meat. Interestingly, the combination of β-alanine and L-histidine supplementation had no significant effect on meat microstructure, porosity, or fat content, indicating that this group showed the best selectivity for improving meat quality. Since increasing the carnosine content without affecting meat texture or enhancing meat quality may be a key factor in improving KR meat characteristics, further studies are required to clarify the effect of carnosine levels on meat processing.

## Figures and Tables

**Table 1:** Effect of supplementation with β-alanine and/or L-histidine on myofiber diameter, distance between myofibers, number of myofibers, myofiber area, and myofiber spacing per cross-section

Treatment	Diameter (μm)	Distance (μm)	Number	Myofiber area (%)	Myofiber spacing (%)
Control	25.85±1.51^b^	37.18±1.12	12.00±2.51	87.66±2.29	12.34±2.29
1.0% β-alanine + 0.5% L-histidine	38.96±2.63^ab^	45.50±6.91	6.67±1.76	85.32±2.43	12.64±1.61
1.0% β-alanine	36.75±2.78^ab^	52.02±7.19	7.00±2.08	86.88±2.14	13.06±2.11
0.5% L-histidine	44.35±4.88^a^	55.93±7.08	5.33±0.88	83.64±1.20	16.36±1.20

a,b Different letters within a column indicate significant differences between the groups (p<0.05).

## SUPPLEMENTARY MATERIAL

**Supplementary Table S1**. Analytical value of amino acids content (%) in experimental diets in different growing phases

**Supplementary Table S2**. Effect of amino acid supplementation on the growth performance of Korat chickens [6]

**Supplementary Table S3**. Carnosine and anserine contents in Korat chicken breast meat [6]
