# Supplementary material for: Effect of precursor amino acids for carnosine synthesis on breast fiber microstructures and myofiber differentiation-related gene expression in slow-growing chicken
Source: Anim Biosci. 2024 Aug 16;37(11):1834–47. doi: 10.5713/ab.24.0012 (PMC11541030; doi:10.5713/ab.24.0012)
Supplement: Supplementary file 1 [file ab-24-0012-Supplementary-Table-1.pdf]

Supplementary Tables

Table S1. Analytical value of amino acids content (%) in experimental diets in different growing phases.

Amino acid	Grower (22 to 42 days)[1]				Finisher (43 to 70 days)[1]			
	A	B	C	D	A	B	C	D
Asp	2.15	1.93	1.96	1.97	1.66	1.76	1.9	1.81
Thr	0.76	0.67	0.67	0.68	0.58	0.60	0.68	0.64
Ser	1.07	0.93	0.90	0.94	0.84	0.85	0.97	0.90
Glu	4.05	3.78	3.82	3.69	3.42	3.49	3.75	3.60
Pro	1.16	1.11	1.03	1.10	1.03	1.03	1.22	1.12
Gly	0.91	0.83	0.82	0.85	0.73	0.76	0.83	0.79
Ala	1.16	1.15	1.13	1.08	1.07	1.04	1.17	1.07
Cys	0.42	0.41	0.35	0.39	0.35	0.41	0.45	0.36
Val	0.97	0.95	0.94	0.94	0.81	0.84	0.93	0.90
Ile	0.84	0.82	0.83	0.82	0.69	0.72	0.79	0.76
Leu	1.76	1.67	1.74	1.72	1.53	1.52	1.71	1.60
Tyr	0.60	0.48	0.46	0.54	0.40	0.46	0.54	0.47
Phe	0.81	0.75	0.78	0.77	0.65	0.68	0.75	0.70
His	0.40	0.44	0.97	1.02	0.30	0.30	0.93	0.97
Lys	1.29	1.08	1.14	1.15	0.96	1.03	1.13	1.09
Arg	1.34	1.19	1.15	1.16	1.01	1.04	1.12	1.11

[1]Treatment groups are A (control), B (supplementation with 1.0% β-alanine), C (supplementation with 0.5% L-histidine), and D (supplementation with 1.0% β-alanine and 0.5% L-histidine), respectively.
