# Supplementary material for: Effect of precursor amino acids for carnosine synthesis on breast fiber microstructures and myofiber differentiation-related gene expression in slow-growing chicken
Source: Anim Biosci. 2024 Aug 16;37(11):1834–47. doi: 10.5713/ab.24.0012 (PMC11541030; doi:10.5713/ab.24.0012)
Supplement: Supplementary file 2 [file ab-24-0012-Supplementary-Table-2.pdf]

Table S2. Effect of amino acid supplementation on the growth performance of Korat chickens (Suwanvichanee et al., 2022).

Parameter	Treatment group[1]				SEM[2]	P-value
	A	B	C	D		
22–42 d						
FI (g)	1018.64	1092.50	1037.32	1038.99	18.67	0.07
BWG (g)	381.97	386.21	382.48	390.17	5.68	0.72
ADFI (g)	48.51	52.02	49.40	49.47	0.89	0.07
ADG (g)	18.19	18.39	18.21	18.58	0.27	0.72
BW 42 d (g)	635.97	643.21	637.98	644.17	6.10	0.74
FCR	2.67	2.83	2.71	2.66	0.05	0.15
43–70 d						
FI (g)	1960.78	1940.13	1964.78	1933.38	59.71	0.98
BWG (g)	512.64	501.04	523.21	562.64	24.05	0.33
ADFI (g)	70.03	69.29	70.17	69.05	2.13	0.98
ADG (g)	18.31	17.90	18.69	20.09	0.89	0.33
BW 70 d (g)	1148.61	1144.25	1161.19	1206.81	26.01	0.34
FCR	3.85	3.91	3.77	3.46	0.19	0.38
22–70 d						
FI (g)	2979.42	3032.63	3002.10	2972.37	68.99	0.93
BWG (g)	894.61	887.25	910.69	952.81	25.65	0.30
ADFI (g)	60.80	61.89	61.27	60.66	1.41	0.93
ADG (g)	18.26	18.11	18.48	19.45	0.52	0.30
FCR	3.34	3.43	3.32	3.13	0.12	0.36

Results were averaged from 25 chickens per replicate, 100 chickens per treatment.

[1]Treatment groups are A (control), B (supplemented with 1.0% β-alanine), C (supplemented with 0.5% L-histidine), and D (supplemented with 1.0% β-alanine + 0.5% L-histidine) respectively.

[2]SEM indicates standard error of mean (mean ± SEM; n = 5/treatment).

FI, feed intake; BWG, body weight gain; ADFI, average daily feed intake; ADG, average daily gain; BW, body weight; FCR, feed conversion ratio.
