# Supplementary material for: Effect of precursor amino acids for carnosine synthesis on breast fiber microstructures and myofiber differentiation-related gene expression in slow-growing chicken
Source: Anim Biosci. 2024 Aug 16;37(11):1834–47. doi: 10.5713/ab.24.0012 (PMC11541030; doi:10.5713/ab.24.0012)
Supplement: Supplementary file 3 [file ab-24-0012-Supplementary-Table-3.pdf]

Table S3. Carnosine and anserine contents in Korat chicken breast meat (Suwanvichanee et al., 2022).

Parameter	Treatment group[1]				SEM[2]	P-value
	A	B	C	D		
Carnosine (µg/g)	2,756.6[c]	3,484.6[b]	3,659.8[b]	4,212.5[a]	82.88	<0.001
Anserine (µg/g)	10,577.2	10,391.6	10,312.7	10,272.8	282.47	0.88

Results were averaged from 10 chickens per treatment.

[a-c] Mean values with different superscripts in the same row indicate significantly different at P-value < 0.05

[1]Treatment groups are A (control), B (supplemented with 1.0% β-alanine), C (supplemented with 0.5% L-histidine), and D (supplemented with 1.0% β-alanine + 0.5% L-histidine) respectively.

[2]SEM indicates standard error of mean
